# Primary hepatic carcinoid tumor: a case report and review of the literature

**DOI:** 10.1186/1757-1626-2-90

**Published:** 2009-01-27

**Authors:** Chih-Wen Lin, Chung-Hsu Lai, Chia-Chang Hsu, Chao-Tien Hsu, Pei-Min Hsieh, Kuo-Chen Hung, Yaw-Sen Chen

**Affiliations:** 1 Division of Hepatogastroenterology, Department of Internal Medicine, E-Da Hospital/I-Shou University, 1, E-Da Road, Jiau-shu Tsuen, Yan-chau Shiang, Kaohsiung county 82445, Taiwan; 2 Division of Infectious Diseases, Department of Internal Medicine, E-Da Hospital/I-Shou University, 1, E-Da Road, Jiau-shu Tsuen, Yan-chau Shiang, Kaohsiung county 82445, Taiwan; 3 Department of Pathology, E-Da Hospital/I-Shou University, 1, E-Da Road, Jiau-shu Tsuen, Yan-chau Shiang, Kaohsiung county 82445, Taiwan; 4 Department of Surgery and Organ Transplantation Center, E-Da Hospital/I-Shou University, 1, E-Da Road, Jiau-shu Tsuen, Yan-chau Shiang, Kaohsiung county 82445, Taiwan

## Abstract

**Background:**

Primary hepatic carcinoid tumor (PHCT) is very rare and difficult to diagnose before biopsy or operation. We report a patient with a small PHCT and review cases in the literature.

**Case presentation:**

A 48-year-old Chinese female with underlying hepatitis B virus (HBV) infection was found to have a low echoic hepatic nodule by abdominal ultrasound. Tumor markers were negative. Dynamic liver computed tomography scans showed enhancement of the nodule in the arterial phase and early washout in the portal phase. Hepatocellular carcinoma (HCC) was considered based on the image findings and underlying HBV infection. However, the tumor biopsy revealed a malignant neoplasm that originating from neuroendocrine cells. Pre-operative and intra-operative investigations for the possible other origin of carcinoid tumor were negative, so PHCT was confirmed.

**Conclusion:**

A small and asymptomatic PHCT is extremely rare. PHCT should be one of the differential diagnoses in patients with small hepatic tumor, even in regions with high prevalence of HBV infection and HCC. Pre-operative biopsy is necessary to avoid misdiagnosis even when HCC is highly suspected clinically.

## Background

Carcinoid tumors are neuroendocrine origin neoplasm and may produce serotonin or other functional peptide hormones. They are well-differentiated and low-grade malignant neoplasms. Approximately 74% of all carcinoid tumors arise from the gastrointestinal (GI) tract and the liver is a common site for metastases [1]. However, primary hepatic carcinoid tumor (PHCT) is very rare and the first case was documented by Edmondson in 1958 [2]. This rarity makes it difficult for clinicians to diagnose accurately before biopsy, resection of tumor, or autopsy [3]. Large-sized tumors are common presentations when they are diagnosed [4]. PHCT presents as a small and single nodule, is extremely rare and is difficult to differentiate from hepatocellular carcinoma (HCC), particularly in a region with high prevalence of HCC. Herein, we report a rare case of small PHCT with underlying HBV infection which was suspected to be a small HCC before operation and also comprehensively review 94 cases of PHCT in the literature.

## Case presentation

**Figure 1:**
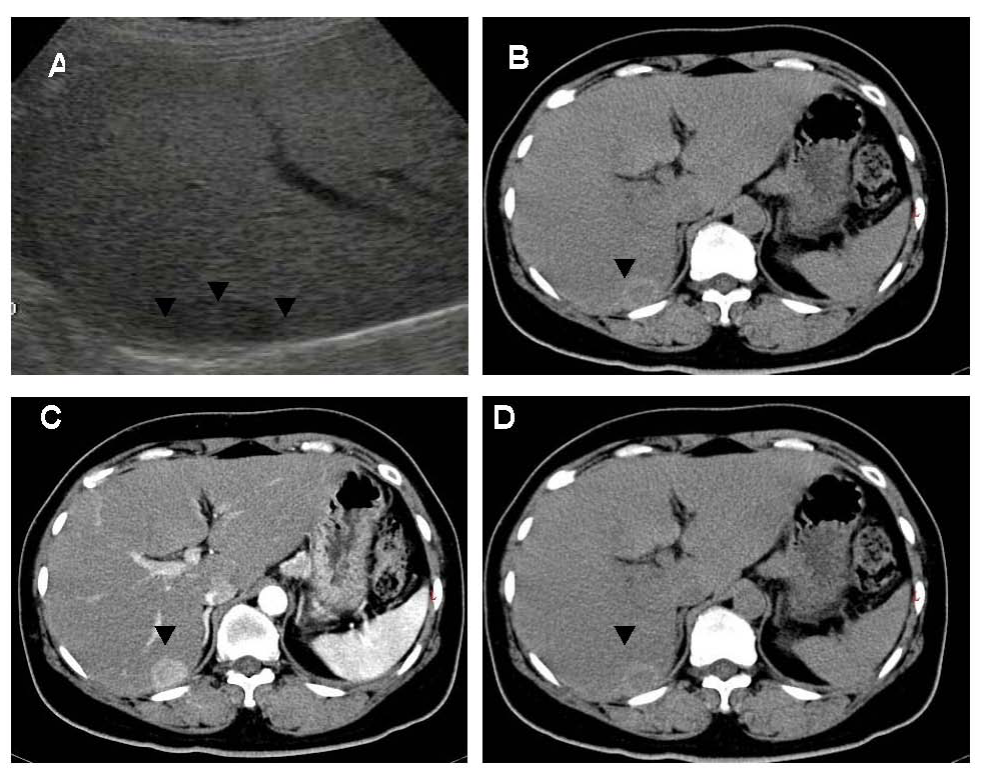
**(A) Abdominal ultrasound shows a low echoic nodule in the sixth segment of the liver (arrowheads)**. **(B) **Noncontrast CT scan shows a well-circumscribed, low density nodule (arrowheads). **(C) **Dynamic CT scan shows enhancement of the nodule in the arterial phase (arrowheads). **(D) **Dynamic CT scan shows enhancement in the portal phase (arrowheads).

**Figure 2:**
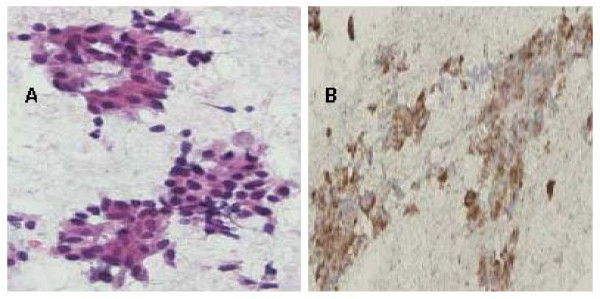
**(A) The tumor was composed of round or ovoid cells arranged in insular pattern with a rosette formation (hematoxylin and eosin)**. **(B) **The tumor cells were positive for chromogranin A.

**Figure 3:**
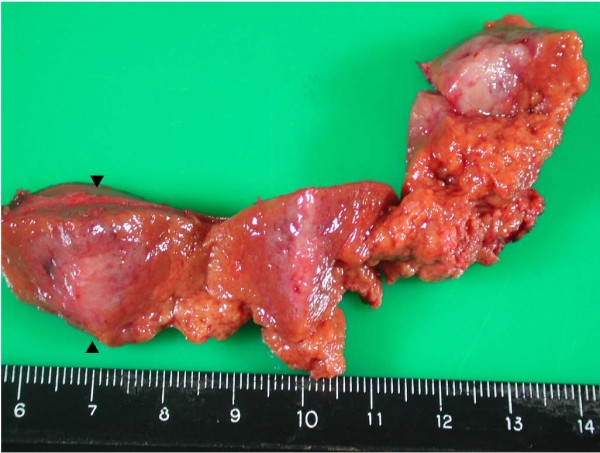
**The resected liver contained a 1.6 × 1.6 × 1.5 cm circumscribed tumor (arrowheads)**.

A 48-year-old Chinese female with HBV infection for 15 years was regularly followed up at gastroenterology outpatient department by abdominal ultrasound (US) and serum alpha-fetoprotein (AFP) screening every six months. In May 2005, abdominal US demonstrated a low echoic nodule, 1.6 × 1.6 cm in size in the sixth segment of the liver and mild parenchymal liver disease (Fig. 1A). She was asymptomatic and no abnormality was disclosed by physical examination. Noncontrast liver computed tomography (CT) showed a 1.6 × 1.6 cm well-circumscribed and low density nodule in the same liver segment (Fig. 1B). Dynamic CT scans showed enhancement of the nodule in the arterial phase and early washout in the portal phase (Figs. 1C and 1D). Serum was positive for hepatitis B surface antigen (HBsAg), but negative for HBeAg. The serum anti-HCV was also negative. AFP and CEA were within normal range. Based on the imaging findings and underlying HBV infection, small HCC was highly suspected and a US-guided liver biopsy was performed for definite diagnosis. However, the pathological finding suggested a malignant neoplasm originating form neuroendocrine cells (Fig. 2A). The result of immunohistochemical (IHC) stain was positive for neuron-specific enolase (NSE), synaptophysin, and chromogranin A (Fig. 2B). The pre-operative 24-hour urine 5-hydroxyindoleacetic acid (5-HIAA) value was within normal limits. We undertook a more thorough investigation to rule out the possibility that the liver tumor was a metastatic carcinoid. This workup included upper and low GI endoscopy, a small bowel series, abdominal US, and chest and abdominal CT scans. All imaging findings were unremarkable. Partial hepatectomy was performed. Small bowel, appendix and large bowel were checked during operation and no tumor was found. No cirrhotic liver was noted. The surgery was performed successfully and the patient recovered well. The resected liver tissue contained a 1.6 × 1.6 × 1.5 cm circumscribed tumor (Fig. 3). Histological examination revealed the round or ovoid neoplastic cells arranged in insular pattern with a rosette formation. There was no tumor embolus in the vessels and the resection line was free from tumor invasion. The IHC stains were positive for chromogranin A, cytokeratin, NSE, and synaptophysin. The final diagnosis was PHCT. She was followed up regularly at our hospital and remained disease-free 3 years after operation.

## Discussion

The origin of PHCT remains unknown. The cells may originate from ectopic pancreatic or adrenal tissue found within the liver or from scattered neuroendocrine cells in the intrahepatic biliary epithelium [5]. Some were observed in animal studies such as those found in the rat liver [6]. The scarcity of such cells may explain the rarity of primary hepatic neuroendocrine tumors. It is also proposed that chronic inflammation in the biliary system may induce intestinal metaplasia that predisposes to the development of neuroendocrine tumor [5].

Including our case, we found 94 cases of PHCT (including primary hepatic neuroendocrine tumor) in the literature [1-5,7-15] and their clinical characteristics are summarized in Table 1. This type of tumor occurs mainly in middle age (mean age, 49.8 years) and is slightly more frequent in females (58.5%). The most common symptom among 84 cases was abdominal pain (37 cases, 44.0%). Eleven cases were asymptomatic (13.1%). The overall mortality rate was 25.5% (24/94) as shown in Table 2.

**Table 1:** Clinical characteristics and imaging findings of 94 cases of primary hepatic carcinoid tumor

Characteristics	Number (%)
Age (y)^a^	49.8 ± 16.0
Gender (M/F)	39/55 (41.5/58.5)
Clinical manifestations	
Asymptomatic	11/84 (13.1)
Abdominal pain	37/84 (44.0)
Carcinoid syndrome	14/84 (16.7)
Diarrhea	8/84 (9.5)
Flushing	4/84 (4.8)
Cushing syndrome	2/84 (2.4)
Abdominal mass	12/84 (14.3)
Fatigue	6/84 (7.1)
Zollinger-Ellison syndrome	5/84 (6.0)
Dyspnea	4/84 (4.8)
Jaundice	4/84 (4.8)
Anemia	2/84 (2.4)
Body weight loss	2/84 (2.4)
Hypoglycermia	1/84 (1.2)
Leg edema	1/84 (1.2)
Imaging finings	
Ultrasound	
Solid tumor	32/39 (82.1)
Solid tumor with cystic component	7/39 (17.9)
Hyperechoic pattern	12/14 (85.7)
Hypoechoic pattern	2/14 (14.3)
Computed tomography	
Low density tumor in noncontrast	37/50 (74.0)
Low density tumor with cystic component in noncontrast	17/50 (34.0)
Tumor enhancement	20/50 (40.0)
Enhanced in arterial phase and washout in portal phase	13/50 (26.0)
Magnetic resonance imaging	
Low intensity in T1 and high intensity in T2	17/21 (81.0)
Angiography	
Hypervascular tumor	23/27 (85.2)
Avascular tumor	4/27 (14.8)

^**a**^Present as mean value ± standard derivation

**Table 2:** Location of tumor, histology and prognosis of 94 cases of primary hepatic carcinoid tumor

Characteristics	Number (%)
Location	
unilobar	72/94 (76.6)
Left lobe	28/94 (29.8)
Right lobe	44/94 (46.8)
billobar	22/94 (23.4)
Number	
Single	59/94 (62.8)
Multiple	35/94 (37.2)
Tumor size	
≦ 3 cm	7/74 (9.5)
> 3 cm	67/74 (90.5)
Histological stains	
Grimelius	64/71 (90.1)
Fontana-Masson stains	14/34 (41.2)
Immunohistochemical stains	
Chromogranin A	57/64 (89.1)
Neuron specific enolase	43/58 (74.1)
Synaptophysin	23/47 (48.9)
cytokeratin	22/40 (55.0)
Misdiagnosed as HCC by microscopy before IHC stains	11/94 (11.7)
Prognosis	
Alive	70/94 (74.5)
Death	24/94 (25.5)
Treatment	
Hepatectomy	73/84 (86.8)
Liver transplantation	3/84 (3.6)
Transcatheter arterial embolization	3/84 (3.6)
Chemotherapy	2/84 (2.4)
Radiotherapy	2/84 (2.4)
Radiofrequency ablation	1/84 (1.2)
Recurrence	
No recurrence	53/75 (70.7)
Recurrence	22/75 (29.3)

HCC: Hepatocellular Carcinoma; IHC: Immunohistochemical;

The case we presented was a small and asymptomatic PHCT. In the literature, only 9.5% (7/74) and 13.1% (11/84) of cases were small-sized tumors (≦ 3 cm) and asymptomatic PHCTs, respectively. Their prognosis was good and the survival rate was 100 % for the 7 small-sized tumor cases and 90.1% (10/11) for the asymptomatic cases, only one of which died as a result of renal cell carcinoma.

The rarity of PHCT makes it difficult to diagnose accurately before biopsy or resection. In previous reports, 4 (4.3%) and 5 (5.3%) cases were diagnosed as HCC [8-10] and cholagiocarcinoma [11,12] respectively by imaging and clinical findings before operation. Moreover, PHCT is also difficult to diagnose because of the histological similarity with HCC [3]. 11.7% (11/94) of cases were misdiagnosed as HCC by light microscopy until they revealed the features of carcinoid tumor by IHC examinations [3,7,9,13]. Furthermore, 6 (6.4%) cases had chronic liver disease with HBV or HCV infection and 3 (3.2%) cases with HBV infection were misdiagnosed as HCC before IHC stains [9,14,15]. Our case had the underlying hepatitis B liver disease and typical imaging presentation of HCC in dynamic CT scans. AFP level may not rise due to small size of tumor. In addition, Taiwan is a region with high prevalence of HBV and HCC. Thus, small HCC was highly suspected before biopsy. Carcinoid tumor was confirmed by liver biopsy and we arranged serial pre-operative and intra-operative examination to investigate the original site of tumor because the liver is a common site for metastases of carcinoid tumors [1]. The accurate diagnosis of carcinoid tumor before operation is very important because it can alert clinicians to seek possible sites of metastasis, thereby avoiding unnecessary operation or a second operation if a metastatic lesion is found.

## Conclusion

A small and asymptomatic PHCT is extremely rare. PHCT should be one of the differential diagnoses in patients with small hepatic tumor, even in regions with high prevalence of HBV infection and HCC. Pre-operative biopsy is necessary to avoid misdiagnosis even when HCC is highly suspected clinically.

## Abbreviations

PHCT: Primary hepatic carcinoid tumor; HBV: Hepatitis B virus; HCC: Hepatocellular carcinoma; GI: Gastrointestinal; US: Ultrasound; CT: Computed tomography; AFP: Alpha-fetoprotein; IHC: Immunohistochemical.

## Consent

Written informed consent was obtained from the patient for publication of this case report, photographic and radiographic images. A copy of the written consent is available for review by the Editor-in-Chief of this journal.

## Competing interests

The authors declare that they have no competing interests.

## Authors' contributions

CL analyzed and interpreted the patient data and was a major contributor in writing the manuscript; CH analyzed and interpreted the patient data and was a major contributor in writing the manuscript; CH performed the histological examination of the liver biopsies and provided the histological images; HP performed the images investigation of the patient and was a major contributor in writing the manuscript; KH performed the images investigation of the patient and was a major contributor in writing the manuscript; CL contributed to the writing and revising of the manuscript; YC contributed to the writing and revising of the manuscript.

All authors read and approved the final manuscript.
